# Altered glycosylation of exported proteins, including surface immune receptors, compromises calcium and downstream signaling responses to microbe-associated molecular patterns in *Arabidopsis thaliana*

**DOI:** 10.1186/s12870-016-0718-3

**Published:** 2016-01-28

**Authors:** Fabian Trempel, Hiroyuki Kajiura, Stefanie Ranf, Julia Grimmer, Lore Westphal, Cyril Zipfel, Dierk Scheel, Kazuhito Fujiyama, Justin Lee

**Affiliations:** 1 Leibniz Institute of Plant Biochemistry, Weinberg 3, D-06120 Halle/Saale, Germany; 2 The International Center for Biotechnology, Osaka University, 2-1 Yamada-oka, Suita-shi, Osaka 565 Japan; 3 Current address: Phytopathology, TUM School of Life Sciences, Weihenstephan, Technische Universität München, Emil-Ramann-Str. 2, D-85350 Freising-Weihenstephan, Germany; 4 Current address: Institute of Biochemistry and Biotechnology, Martin Luther University Halle-Wittenberg, Weinbergweg 22, D-06120 Halle, Germany; 5 The Sainsbury Laboratory, Norwich Research Park, Norwich, NR4 7UH UK

**Keywords:** Calcium, Immune signaling, Pattern recognition receptors (PRRs), *N*-glycosylation, Defense

## Abstract

**Background:**

Calcium, as a second messenger, transduces extracellular signals into cellular reactions. A rise in cytosolic calcium concentration is one of the first plant responses after exposure to microbe-associated molecular patterns (MAMPs). We reported previously the isolation of *Arabidopsis thaliana* mutants with a “*changed calcium elevation*” (*cce*) response to flg22, a 22-amino-acid MAMP derived from bacterial flagellin.

**Results:**

Here, we characterized the *cce2* mutant and its weaker allelic mutant, *cce3*. Besides flg22, the mutants respond with a reduced calcium elevation to several other MAMPs and a plant endogenous peptide that is proteolytically processed from pre-pro-proteins during wounding. Downstream defense-related events such flg22-induced mitogen-activated protein kinase activation, accumulation of reactive oxygen species and growth arrest are also attenuated in *cce2/cce3*. By genetic mapping, next-generation sequencing and allelism assay, *CCE2/CCE3* was identified to be *ALG3* (*Asparagine-linked glycosylation 3*). This encodes the α-1,3-mannosyltransferase responsible for the first step of core oligosaccharide Glc_3_Man_9_GlcNAc_2_ glycan assembly on the endoplasmic reticulum (ER) luminal side. Complementation assays and glycan analysis in yeast *alg3* mutant confirmed the reduced enzymatic function of the proteins encoded by the *cce2/cce3* alleles – leading to accumulation of M5^ER^, the immature five mannose-containing oligosaccharide structure found in the ER. Proper protein glycosylation is required for ER/Golgi processing and trafficking of membrane proteins to the plasma membrane. Endoglycosidase H-insensitivity of flg22 receptor, FLS2, in the *cce2/cce3* mutants suggests altered glycan structures in the receptor.

**Conclusion:**

Proper glycosylation of MAMP receptors (or other exported proteins) is required for optimal responses to MAMPs and is important for immune signaling of host plants.

**Electronic supplementary material:**

The online version of this article (doi:10.1186/s12870-016-0718-3) contains supplementary material, which is available to authorized users.

## Background

For optimal growth, plants need to fend off pathogen attacks. Besides preformed barriers, one of the initial resistance mechanisms relies on the sensing of conserved microbe- or pathogen-associated molecular patterns (MAMPs/PAMPs) or of endogenous plant molecules released during the infection process (so-called damage-associated molecular patterns, DAMPs). This activates signaling events to coordinate local and systemic defense responses and mount so-called pattern-triggered immunity (PTI) [1]. MAMPs recognized by plants include microbe cell wall components such as lipopolysaccharides (LPS) [2], peptidoglycan [3] or chitin [4–8] or bacterial proteins such as flagellin [9] or EF-Tu [10], while DAMPs could be extracellular ATP [11], oligogalacturonides released from pectin [12] or plant peptides known as PEPs [13].

Sensing of MAMPs/DAMPs is achieved after binding to their cognate pattern-recognition receptors (PRRs), which are typically surface-localized proteins with an extracellular ectodomain for ligand-binding, a single-pass transmembrane domain and in some cases, intracellular signaling domains (see below) [14]. Different ectodomains (such as LysM, leucine-rich repeats (LRR), lectin and epidermal growth factor (EGF)-like domains) correspond to the different classes of molecules they bind (for a comprehensive review see [15]). In plants, most of the LRR-type receptors recognize peptidic MAMPs, whereas known LysM-, lectin- and EGF-like domain containing receptors bind different (lipo)glycan structures [16]. PRRs with an intracellular kinase domain are designated as receptor-like kinases (RLKs) while those lacking such signaling domain are classified as receptor-like proteins (RLPs) [17]. Both RLKs and RLPs form heterocomplexes with co-receptors or adapter proteins for full induction of immune signaling. Examples of such protein partners include members of the SERK (somatic embryogenesis receptor kinase) family and/or SOBIR1 (suppressor of BIR1) [18]. For LysM-domain PRRs such as CERK1 (chitin elicitor receptor kinase 1), two interacting kinases, LIK1 [19] and LYK5 [20], regulate chitin signaling. Contrary to earlier work showing chitin-induced CERK1 dimerization to form the optimal chitin-binding pocket [21], LYK5-CERK1 heterodimer is now thought to be the major chitin receptor with a higher affinity than CERK1 homodimers [20].

Upon MAMP/DAMP binding to the receptors, *cis* and *trans* phosphorylation between the receptor complex components [9] and phosphorylation of other intracellular targets are required for immune responses. One class of intracellular phosphorylation targets of PRR complexes are the receptor-like cytoplasmic kinases (RLCKs) such as BIK1 (Botrytis-induced kinase 1) and other members of the BIK1/PBL (avrPphB sensitive 1-like) family such as PBL1, PBL2 and PBL5, as well as BSK1 (Brassinosteroid receptor-signaling kinase1) [22–25]. These RLCKs, which are released from the receptor complex upon phosphorylation, can in turn phosphorylate downstream substrates. The NADPH oxidase RBOHD (respiratory burst oxidase homolog D), which is the main source of apoplastic reactive oxygen species (ROS) upon MAMP perception [26, 27], has been recently described as a target of BIK1, directly linking pattern recognition with a defense and signaling output [28, 29]. RBOHD is also targeted by the calcium dependent protein kinase, CPK5 [30], thus showing an interplay of ROS, phosphorylation and calcium signaling.

As integral membrane proteins, PRRs are subjected to endoplasmic reticulum (ER)-based quality control and the relevance of asparagine-linked glycosylation (*N*-glycosylation) for receptor function and PTI has been shown [31–34]. *N*-Glycosylation, the transfer of oligosaccharide moieties onto asparagine residues of proteins targeted to the secretory pathway, is a conserved protein modification [35]. In eukaryotes, this is achieved co- and post-translationally by the oligosaccharyl transferase (OST) complex in the ER lumen, which transfers a pre-formed tetradecasaccharide (the so-called core oligosaccharide) to the nascent polypeptide as it is being transferred through the translation translocon. The structurally conserved core oligosaccharide is pre-assembled on a dolichol-pyrophosphate (Dol-PP) carrier at both faces of the ER membrane by a series of glycosyltransferases termed ALGs (Asparagine-Linked Glycosylation). For a summary of the pathway, please see Additional file 1: Figure S1. Synthesis starts at the cytoplasmic face of the membrane by the sequential transfer of *N*-acetylglucosamine (GlcNAc) and mannose (Man) residues to the growing glycan structure. After flipping through the membrane, the final synthesis steps are carried out at the luminal face of the ER membrane by ER-localized ALGs through the transfer of Man and glucose (Glc) residues. The finished glycan (Glc_3_Man_9_GlcNAc_2_) is then transferred *en bloc* to growing polypeptides by the OST complex [36]. *N*-Glycosylation affects biological functions of the modified protein; one of which is in the ER quality control of protein folding during the calnexin/calreticulin (CNX/CRT) cycle. Briefly, unfolded proteins undergo a cycle of glycan trimming or reglycosylation by ER glucosidases or UDP- Glucose:glycoprotein glucosyltransferase, respectively, facilitating binding or dissociation with either CNX or CRT, which are lectins specific for monoglucosylated core oligosaccharides. This is repeated until proteins are correctly folded, whereby a final glycan trimming step releases the protein from recognition by CNX and CRT and allows the protein to exit the ER to the Golgi complex. If the protein remains unfolded, it is targeted for ER-associated degradation (ERAD) [37]. Therefore, *N-*glycosylation is important for the quality control of all secreted and membrane proteins. In the case of MAMP signaling, plants with non-functional calreticulin 3 (CRT3) or OST subunits accumulate the EF-Tu-receptor EFR at lower levels than the wild type and are more susceptible to infection by *Pseudomonas syringae* pv. tomato (*Pto*) DC3000. Surprisingly, function and protein levels of the FLS2 receptor appear to be less affected in *crt3* and *ost* mutants [32, 34, 38].

After PRR activation, ROS and nitric oxide generation, MAPK activation, as well as ion fluxes (including an increase in cellular calcium concentration) constitute the early signaling steps that coordinately control MAMP/DAMP signal transduction. The molecular identity of the plasma-membrane calcium channels responsive to MAMPs/DAMPs is still unclear [18]. Isolation of calcium signaling mutants has suggested a very close association between receptor complexes and calcium (in)flux-mediating channels [39]. Although no direct interaction has been shown to date, genetic and inhibitor-based studies suggest channels may be activated by phosphorylation through the PRR complex components or their downstream target(s) [24]. Alternatively, as was proposed for the Pep1 (DAMP) receptor(PEPR1), a guanylyl cyclase activity of PEPR1 generates cGMP to activate the CNGC2 cyclic nucleotide gated calcium channel [40]. The Arabidopsis Ca^2+^-ATPase ACA8, which regulates MAMP responses, directly interacts with the flagellin receptor, FLS2 [41]. These findings support the hypothesis of close association and tight integration of calcium signaling with the receptor complexes in plant immune signaling.

To elucidate the MAMP-mediated calcium signaling pathway, we previously described the isolation of several mutants with a *changed calcium elevation* (*cce*) phenotype using the flagellin-derived flg22 peptide. Besides several new *fls2* and *bak1* (*BRI1 receptor associated receptor kinase 1*) alleles, *cce* mutants with enhanced or reduced calcium elevations were identified [39]. In this work, we show that the allelic *cce2* and *cce3* mutants are caused by mutations in *AtALG3 (Asparagine-Linked Glycosylation 3)*, thereby resulting in underglycosylation of several PRRs and compromised MAMP/DAMP signaling.

## Results

### Genetic analysis of the two *changed calcium elevation* mutants, *cce2* and *cce3*

**Fig. 1:**
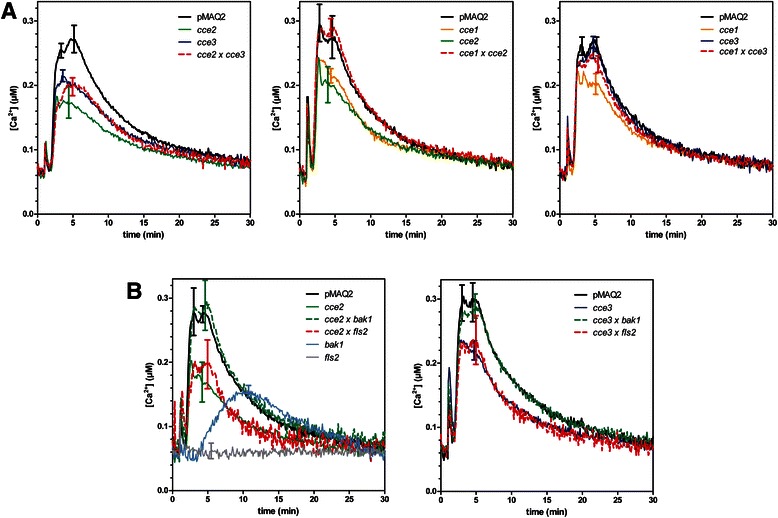
Allelic test of three *cce* mutants. **a** Flg22-induced calcium elevations were measured in 8-day-old seedlings of the F1 generation of the indicated crosses between the *cce1, cce2 and cce3* mutants and compared to the parental lines. **b** *cce2* or *cce3* were crossed to *bak1-4* or *fls2* (SALK_062054) and flg22-induced calcium elevations determined as described above. Traces for the F1 crosses are always marked with *broken lines*. Error bars denote standard deviations (*n* > 10). pMAQ2 is the corresponding aequorin-expressing parental line of the *cce* mutants

We tested three of the previously reported mutants with reduced calcium elevation after flg22 treatment [39] for allelism. The lack of complementation in the F1 seedlings of the crosses between the *cce2* and *cce3* mutants indicates that these are allelic. By contrast, the *cce1* mutant complemented *cce2* or *cce3* and is mutated in a different gene (Fig. 1a). For the rest of this report, we will concentrate on the characterization of the *CCE2*/*CCE3* locus.

**Fig. 2:**
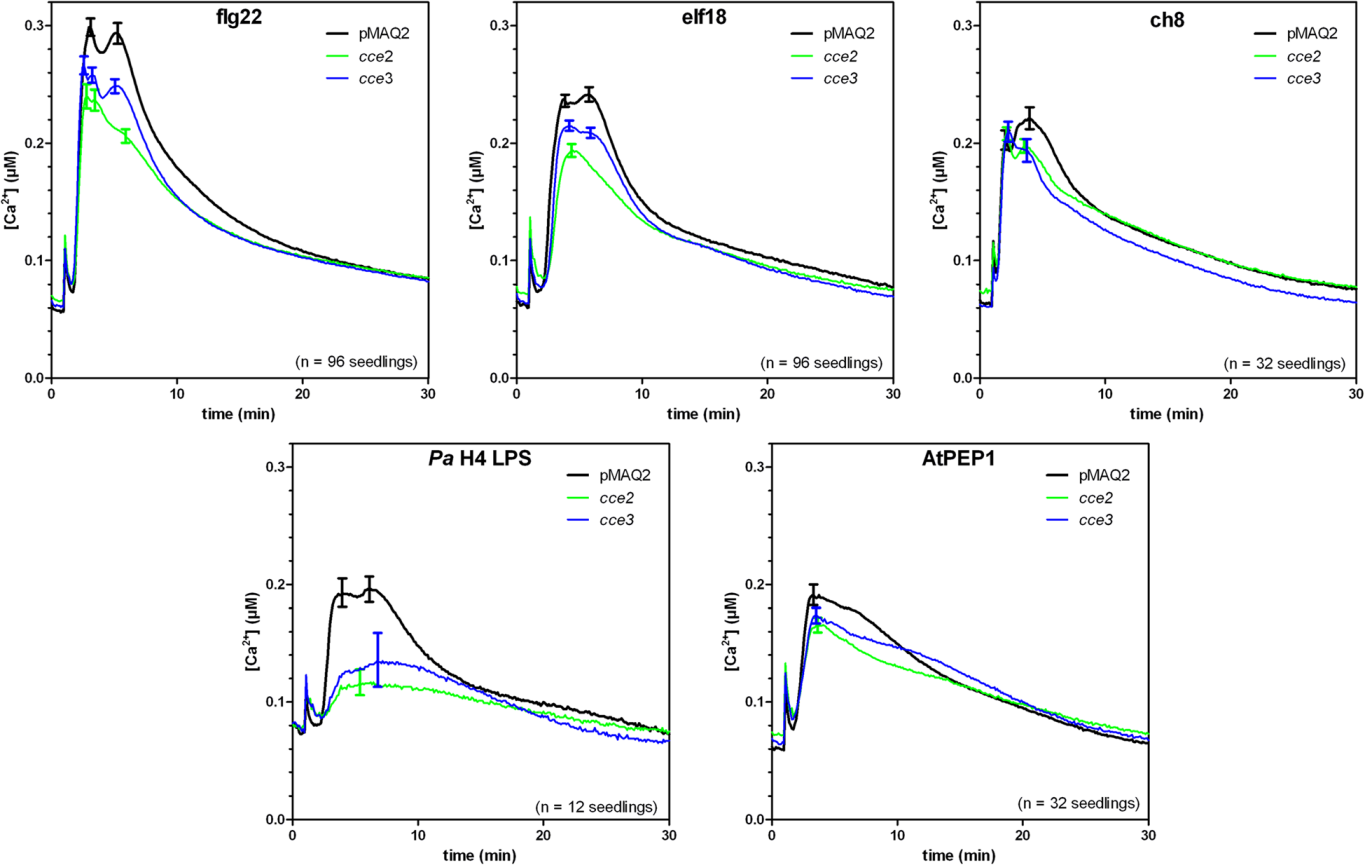
Calcium elevations induced by various MAMP/DAMPs. Eight-day-old seedlings were treated with 1 μM of the indicated peptide MAMPs, DAMP or chitin octamer (ch8) or 25 μg/ml lipopolysaccharide prepared from *Pseudomonas aeruginosa* strain H4 (*Ps* H4 LPS). Error bars depict the 95 % confidence intervals

Further crosses were made with *bak1* or *fls2* mutants to exclude that *cce2*/*cce3* are weak mutant alleles of known flg22 signaling components that have been shown to affect calcium elevations. In the F1 crosses between *bak1* and *cce2* or *cce3*, the flg22-induced calcium elevation was restored to levels similar to that in the parental pMAQ2 line (Fig. 1b). By contrast, the cross with *fls2* did not complement the *cce2/cce3* phenotype. While an initial inference of this observation would be that *cce2*/*cce3* is in fact mutated in *FLS2*, no mutations was detected in the sequence of a 4031 bp amplicon of the *FLS2* gene from *cce2/cce3* mutants [39]. In addition, the reduced calcium elevations in the *cce2/cce3* mutants are also seen after treatment with other MAMPs/DAMPs such as elf18, chitin, LPS and AtPep1 (Fig. 2). This broad spectrum effect of the *cce2/cce3* mutation to multiple MAMPs/DAMPs argues against a mutation in a specific PRR gene but for a common factor needed for receptor function. Also noteworthy from these measurements is that the reduction in MAMP/DAMP-induced calcium elevation is more pronounced for *cce2* than *cce3* (Fig. 2), indicating that *cce3* is a weaker mutation compared to *cce2*.

### Altered flg22-induced responses in the *cce2/cce3* mutants

**Fig. 3:**
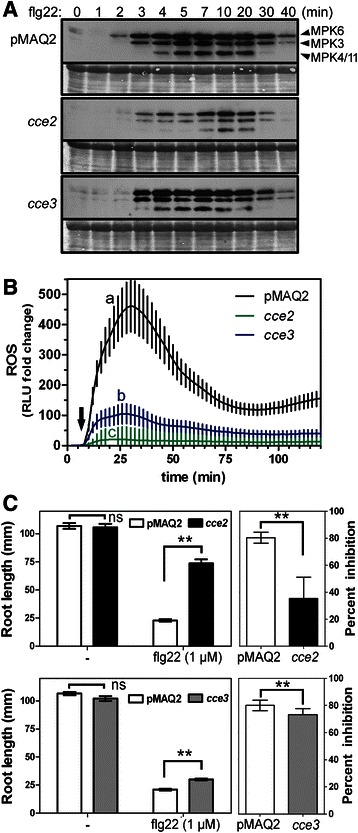
Some flg22-induced responses downstream of calcium transients are partially attenuated **a** Fourteen-day-old seedlings were collected at the indicated time points after flg22 (100 nM) treatment and proteins extracted for western blot analysis with an antibody that recognizes phosphorylated (i.e. activated forms of) MAPKs. The identities of the three MAPK bands are marked on the right. The experiment was repeated thrice with similar outcome. **b** Reactive oxygen species (ROS) accumulation was assessed in 24 leaf disks per genotype over 120 min. The basal luminescence (relative light units, RLU) of each disk was monitored for 6 min (before treatment) and used to normalize the fold change in RLU after flg22 treatment (marked with *arrow*). Error bars depict the 95 % confidence intervals. Statistical significance was analyzed using one-way ANOVA with Tukey’s multiple comparison post-test (The different alphabets denote groups that are considered statistically different, *p* < 0.05). **c** Growth inhibition of *cce2/cce3* mutants compared to the pMAQ2 parental line. Seedlings were placed on agar media without (−) or containing flg22 (1 μM) and the root length measured after 14 days. Left panels show the absolute root lengths of a representative experiment (*n* = 22–23 seedlings). Right panels are the percent inhibition consolidated from 3 independently performed experiments (*n* = 67–69 seedlings). Statistical significance was analyzed using two-way ANOVA with Bonferroni post-test (** = *p* < 0.0001, ns = not significant)

To test how other responses, in addition calcium elevations, are affected in the *cce2/cce3* mutants, we looked at flg22-induced MAPK activation, reactive oxygen species (ROS) accumulation and growth arrest. Based on an immunoblot assay that detects phosphorylated (i.e. activated) forms of MAPKs, the flg22-induced MAPK activation is delayed and reduced in *cce2* (Fig. 3a). For *cce3*, while there is little difference in the activated MAPK band intensities, there appears to be a marginal delay in the timing of MPK6 activation (that can be seen in three independent experiments). Flg22-stimulated ROS accumulation is another rapid plant response that can be measured within minutes after MAMP exposure. In agreement with the attenuated calcium response, the relative ROS accumulation is reduced in the *cce2/cce3* mutants compared to the parental pMAQ2 line (Fig. 3b).

Plants constantly exposed to MAMPs are typically stunted in growth. Based on root length differences of seedlings grown without or with flg22 (1 μM), it can be seen that the *cce2* mutants are less inhibited than the pMAQ2 parents (Additional file 1: Figure S2). By comparison, the primary roots of the *cce3* mutants are still strongly reduced in length when grown on flg22-containing media but are, nevertheless, significantly (*p* < 0.01) longer than the pMAQ2 parent. This growth arrest by flg22 is dependent on the presence of a functional FLS2 receptor [39]. A representative experiment of three independent experiments is shown in Fig. 3c (left panels). While there are variations in absolute root lengths between experiments, the percent growth inhibition consolidated from the three independent experiments (Fig. 3c, right panels) confirm that *cce3* is less affected than the *cce2* mutant. Taken together, all three tested flg22-induced reactions show attenuated responses in the *cce2*/*cce3* mutants. Additionally, in agreement with the MAMP/DAMP-induced calcium elevation, the less dramatic impact of the *cce3* allele supports the notion that *cce3* is a weaker allele compared to *cce2*.

### Mapping and next generation sequencing data reveal SNPs in At2g47760

To identify the *CCE2* gene, the stronger *cce2* mutant was crossed to the Arabidopsis accession Landsberg *erecta* (L*er*-0). The mapping analysis of 44 F2 plants suggested that *CCE2* is located on chromosome 2 between the INDEL marker CER452347 [42] at 18892029 bp (TAIR 10, 5 recombinants) and the end of the chromosome at 19698289 bp (TAIR 10). This region contains 233 gene loci. Illumina whole genome sequencing was performed with genomic DNA prepared from the *cce2* mutant as well as from the pMAQ2 parental line. Comparison to the reference Col-0 genome revealed a total of 405 tentative single nucleotide polymorphisms (SNPs) in *cce2*, of which 77 SNPs led to missense mutations (Additional file 2: Table S1). Within the mapped region on chromosome 2, two SNPs (in gene loci At2g46060 and At2g47760) were detected, which could be re-confirmed by sequencing DNA amplicons encompassing the mutated regions from *cce2*. Since *cce3* is allelic to *cce2*, the corresponding *cce3* genomic region was also PCR-amplified and sequenced. No SNP was found for At2g46060 but a SNP was detected for the locus At2g47760 in the *cce3* amplicons. At2g47760 encodes ASPARAGINE-LINKED GLYCOSYLATION 3 (ALG3), the dolichol pyrophosphate (Dol-PP)-mannose α-1,3-mannosyltransferase that catalyzes the first mannosylation of precursor glycans after flipping of the glycans from the cytosolic to the luminal side of the ER (See Additional file 1: Figure S1 for biosynthesis pathway) [43, 44].

**Fig. 4:**
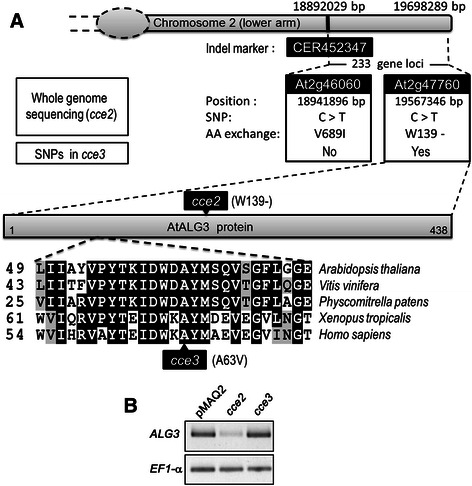
The *cce2/cce3* mutants contain SNPs in the gene locus *At2g47760* (*ASPARAGINE-LINKED GLYCOSYLATION 3, ALG3*). **a** Schematic representation summarizing the SNPs from whole genome re-sequencing of *cce2* within the relevant lower arm of chromosome 2 identified by genetic mapping and amino acid (AA) exchanges in the predicted protein sequences of the *cce2/cce3* alleles. CER452347 is the indel marker used for mapping the *cce2* mutation onto the lower arm of chromosome 2. Sequence alignment of a segment of the ALG3 homologs from the indicated organisms is depicted to highlight that the amino acid exchange caused by the *cce3* mutation lies in a conserved region. **b** Reverse-transcription-PCR analysis of the *ALG3* transcripts. *Elongation factor 1-α* (*EF1-α*) was used as a loading control

The *cce2* allele is a nonsense mutation that converts tryptophan 139 to a premature stop while the *cce3* SNP leads to an A63V exchange. Semi-quantitative reverse-transcription-PCR showed that transcripts for the *cce2/cce3*-derived mRNAs were detectable; although, presumably due to nonsense-mediated mRNA decay, the expression in the *cce2* background was much lower (Fig. 4b).

### Allelism assay by crossing with a T-DNA insertion mutant (*alg3-T*)

**Fig. 5:**
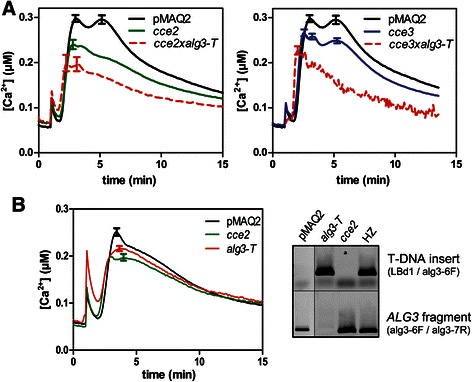
The *cce2*/*cce3* mutants are allelic to *ALG3*. **a** A T-DNA insertion *alg3* mutant (*alg3-T,* SALK_064006) was crossed to *cce2* and *cce3,* successful crosses were validated by PCR in the F1, and the flg22-induced calcium response was monitored in the segregating F2 seedlings (marked with *red broken lines*). Error bars denote standard deviations (n =96 for pMAQ2, *cce2* or *cce3*; *n* = 66 for *cce2*x*alg3-T*, *n* = 49 for *cce3*x*alg3-T*). The same pMAQ2 (“wild type”) reference calcium curve was used for both graphs. Calcium signatures were tested for statistical significance between genotypes by one-way ANOVA (with Tukey’s multiple comparison test, *p* < 0.05). **b** After identifying homozygous *alg3-T* individuals (from the *cce2*x*alg3-T* cross with the indicated primer pairs by PCR of genomic DNA, right panel), flg22-induced calcium response was monitored as above except leaf disks (Ø 4 mm) of 4-week-old plants were used (*n* = 96 for *alg3-T*; n =32 for pMAQ2 or *cce2*). Calcium elevations in *cce2* and *alg3-T* are not different but are both statistically distinct from the pMAQ2 control (one-way ANOVA with Tukey’s multiple comparison test, *p* < 0.01)

To confirm that the detected mutations in *ALG3* are causal for the *cce2/cce3* phenotype, crosses were made with a T-DNA *alg3* insertion mutant, *alg3-T* [44]. The success of the cross was confirmed by PCR in the F1 generation and the calcium fluxes tested in the F2 generation. If *ALG3* is not responsible for the reduced calcium signature in *cce2/cce3*, one would expect 75 % of the segregating F2 population to have a wild type signature. However, there is no complementation and the “calcium response” in the F2 population is reduced in amplitude compared to the pMAQ2 control (Fig. 5a). This suggests that *alg3-T* and *cce2/cce3* are allelic. Furthermore, we genotyped F2 plants from the *cce2* x *alg3-T* cross to identify aequorin-expressing plants that are homozygous for the *alg3-T* mutation and leaf disks from these plants were directly used for calcium measurements. As seen in Fig. 5b, flg22-induced calcium elevation in *alg3-T* is reduced compared to the pMAQ2 controls but not significantly different from the *cce2* (1-way ANOVA, *p* < 0.01).

### The *cce2/cce3* alleles fail to or only partially complement a yeast *alg3* mutant

**Fig. 6:**
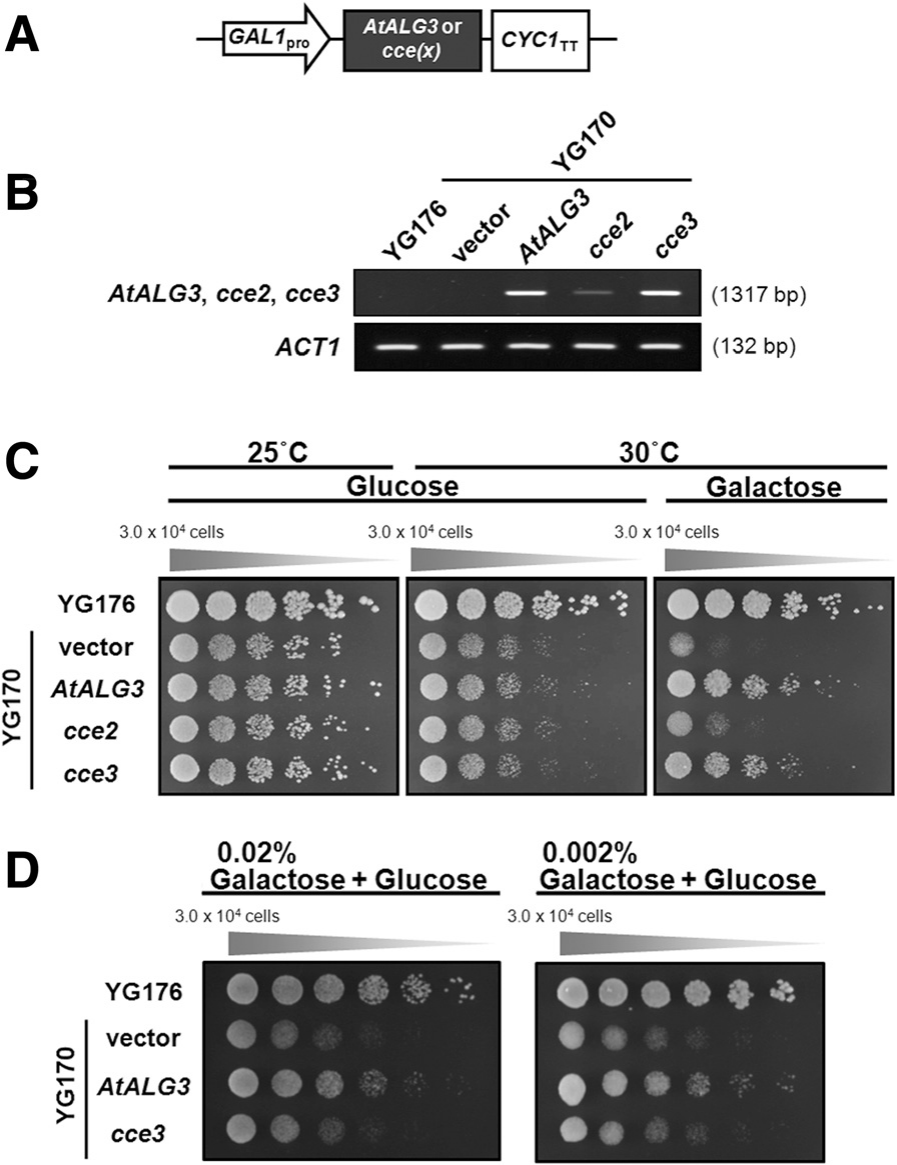
Complementation of yeast *alg3-1* mutant strain (YG170) with the Arabidopsis *ALG3* variants. The Arabidopsis *ALG3* (*AtALG3*) open-reading frames (ORFs) were amplified from cDNAs of wild-type plants or the *cce2/cce3* mutants, cloned into yeast expression vectors, and transformed into yeast strain YG170. **a** Scheme of expression vector constructs of the indicated ORFs under the control of a galactose-inducible promoter (GAL1_pro_) and a cytochrome c terminator (CYC1_TT_). **b** RT-PCR validation of gene expression from the transfected constructs. *Actin 1* (*ACT1*) was used as an amplification/loading control. **c** Complementation of the *alg3-1* mutant yeast strain YG170 was determined by comparing growth without or with galactose-induced expression of the indicated constructs under non-permissive temperature (30 °C). Growth at permissive temperature (25 °C) was used to show the viability of the yeast cells. Five serial dilutions were performed in all cases. **d** Complementation was further tested by reducing expression of the *AtALG3* or the *cce3* variant (by decreasing galactose concentrations from 2 % to 0.02/0.002 %)

The A63V amino acid exchange in ALG3 protein encoded by the *cce3* allele lies in a conserved region of ALG3 proteins of several taxa, ranging from lower/higher plants, amphibians to mammals (Fig. 4). However, alanine to valine is a “conservative” amino acid exchange. Thus, we were interested to see if this mutated variant is functional. Therefore, we cloned the ORFs from the *cce2* and *cce3* alleles and used them to complement a *Saccharomyces cerevisiae alg3*-deficient mutant strain (YG170) [44, 45]. These are under the control of a galactose-inducible promoter and reverse-transcription-PCR analysis showed that these are well expressed in the yeast cells (Fig. 6a, b). Under restrictive conditions (at 30 °C), the *alg3*-deficient strain (YG170) does not grow when transformed with an empty vector or with the *cce2*^*W139stop*^ allele, but is able to grow when expressing wild type *AtALG3* or the *cce3*^*A63V*^ variant, albeit a little less growth is seen for the latter (Fig. 6c). When the galactose concentration used for inducing expression is lowered, reduced complementation is seen for *cce3*^*A63V*^ (Fig. 6d). These results suggest that the ALG3^A63V^ variant is enzymatically functional but is possibly less efficient.

### Yeast glycan analysis confirms the enzymatic activity of ALG3

**Fig. 7:**
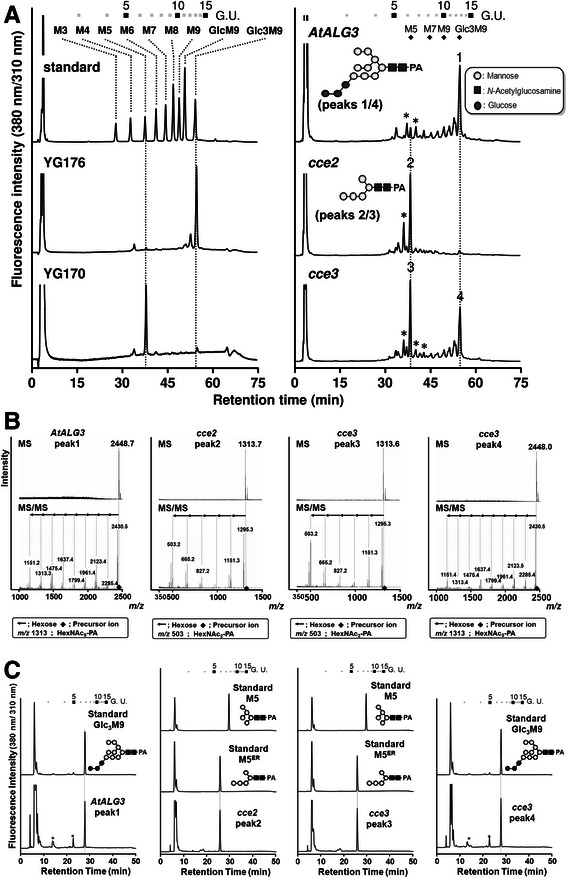
Analysis of dolichol-linked oligosaccharides. **a** HPLC-based size fractionation of 2-pyridylamine (PA)-labelled oligosaccharides after acid hydrolysis and purification of the lipid-linked oligosaccharides from the indicated yeast strains. Inserts depict the deduced structures of peaks 1/4 and peaks 2/3 (as described below in **b** and **c**). **b** Structure determination by LC-MS/MS of peaks 1–4. **c** RP-HPLC comparison of peaks 1–4 to known oligosaccharide standards. Symbols depicting oligosaccharide structures are as defined in A. *For A and C, low levels of contaminants in the HPLC traces are marked with *asterisks*. (Abbreviations: G.U. = Glucose oligomer unit, Glc = Glucose, GlcNAc = *N*-Acetylglucosamine)

To confirm that the yeast growth is due to complementation of the *alg3* deficiency in the yeast strain YG170, lipid-linked oligosaccharides were extracted from the various yeast strains grown in galactose-inducing media, hydrolyzed and labelled with 2-pyridylamine (PA) for glycan analysis. HPLC-based size fractionation showed distinct major peaks with retention times matching those of the Glc_3_M9 standards in the wild type (YG176) and the complemented strain. The *alg3* mutant (YG170) and the *cce2-*complemented strains both have a major peak with retention time that corresponds to the 5-mannose-containing M5 standard. The *cce3*-complemented strain had both peaks (Fig. 7a).

Four samples (peak1 from the *AtALG3* complemented strain; peak2 from the *cce2-*complemented strain; and peaks 3 and 4 from the *cce3-*complemented strain; see Fig. 7a) were collected. Structural determination by LC-MS/MS (Fig. 7b) indicated peaks1/4 to be Glc_3_Man_9_GlcNAc_2_-PA (or Hex_12_HexNAc_2_) while peaks 2/3 to be M5^ER^ (i.e. the 5-mannose-containing Man_5_GlcNAc_2_-PA substrate of ALG3 found in the ER; or Hex_5_-PA). When rechromatographed by reverse-phase-HPLC, the elution time of peak 1 matched the Glc_3_M9 PA-sugar standard while peaks 2/3 differed from the M5 PA-sugar standard, which is the form typically generated in the eukaryotic Golgi apparatus, but co-migrated with the M5^ER^ form (Fig. 7c). These findings confirm that the complemented AtALG3 or the ALG3^A63V^ variants are indeed ER-localized α-1,3-mannosyltransferases in vivo*.* The *cce3*^*A63V*^*-*complemented yeast contained a mixture of both Glc_3_M_9_GlcNAc_2_ and M5^ER^ forms, suggesting that the ALG3^A63V^ protein has reduced enzyme activity or alternatively, it may be partially mis-localized in the yeast cells.

### Various plant pattern recognition receptors are underglycosylated but appear to be still targeted to the plasma membrane

**Fig. 8:**
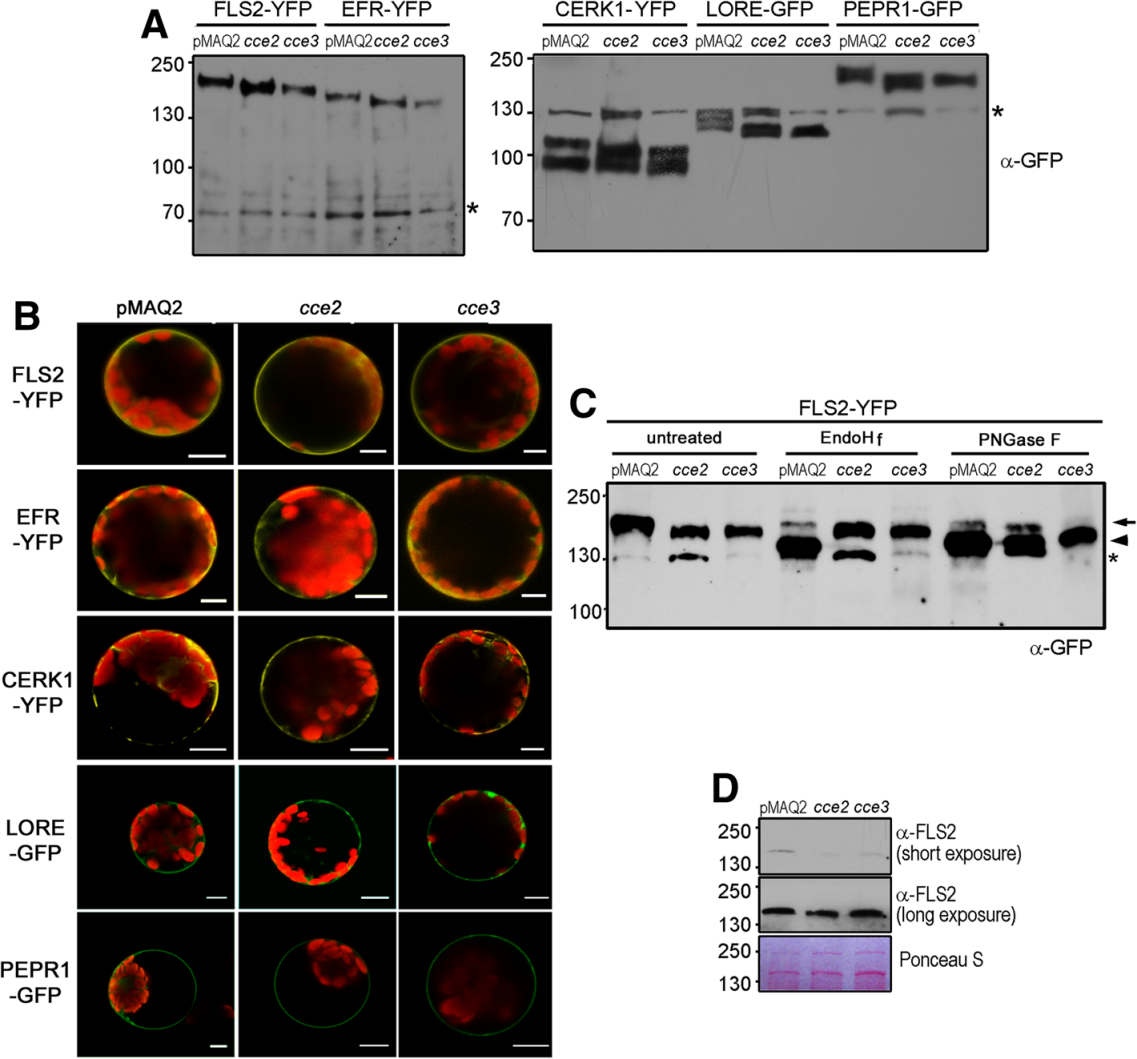
Glycosylation and localization of PRRs in the *cce2/cce3* mutants. **a** Plasmid constructs for expressing *p35S::PRR-YFP/GFP* fusions were transfected into protoplasts isolated from the indicated genotypes and analyzed by western blotting with an α-GFP antibody. The *asterisks mark* unspecific bands recognized by the GFP antibody, which was used as an internal loading control. Note that different buffers (see methods section) were used for protein extraction in the right or left panels. Numbers on the left are the molecular size markers in kDa. **b** Confocal microscopical images of the GFP- or YFP- tagged PRRs in the pMAQ2 parental, *cce2* or *cce3* background. Arabidopsis protoplasts were transiently transfected with plasmids for expressing the indicated PRRs. Scale bars represent 10 μm. **c** FLS2-GFP was expressed in protoplasts as described above in A, and extracted membrane proteins were subjected to endoglycosidase H (EndoH_f_) or peptide:*N-*glycosidase F (PNGase F) digestion. An *arrow marks* the weak glycosidase-resistant FLS-YFP bands while the *arrowhead marks* the mobility shift of the digested proteins. In the *cce* mutants, Endo Hf-resistance results from altered *N*-glycan structure upon blockage of the *ALG3* step in the pathway (see Additional file 1: Figure S1). In the PNGase-F digests, remaining weak bands of FLS2-YFP in both wildtype (pMAQ2) and *cce* lanes may be explained by about 10 % core fucose-decorated (and thus PNGase F-resistant) *N*-glycans with terminal GlcNAc residues (see Table II of Kajiura et al. [44]), indicative of passage through the Golgi apparatus after correct folding of FLS2-YFP in the ER. Note that the ~130 kDa bands (marked with *asterisk*) are unspecific signals that appear with the anti-GFP antibody. **d** Endogenous FLS2 levels in the *cce2/cce3* mutants were compared to the pMAQ2 parental line. Western blotting (α-FLS2) was used to visualize FLS2 levels in microsomal proteins prepared from 8-day-old seedlings

Since glycosylation is important for the ER quality control of PRRs [31, 32, 34], we analyzed the PRR proteins in the *cce2/cce3* mutants by transiently expressing YFP/GFP-tagged PRRs in Arabidopsis protoplasts. As expected for compromised *N-*glycosylation, a higher mobility (in SDS-PAGE) of the tagged FLS2, EFR, CERK1, LORE (putative LPS receptor) and PEPR1 was observed when expressed in the *cce2/cce3* background as compared to the parental pMAQ2 (Fig. 8a). The respective MAMPs/DAMPs all elicited a reduced calcium response in the *cce2/cce3* background (Fig. 2). To know if this may be due to altered PRR localization, confocal microscopy was used to image the YFP/GFP-tagged PRRs. In agreement to plasma membrane localization, most of the YFP/GFP signals were at the periphery, and in some cases, in “patchy” spots along the membrane, of the protoplasts. No obvious difference in localization was seen between the mutants and the pMAQ2 background (Fig. 8b).

To check if the PRRs are indeed glycosylated, we treated the membrane protein extracts of the transfected protoplasts with peptide-*N-*glycosidase F (PNGase F) to remove the *N*-glycan chains directly from the asparagine. FLS2 was used as a representative PRR for our assay. Most of the FLS2-YFP were digested and showed a mobility shift after PNGase F treatment for all the tested genotypes. This indicates that the FLS2 protein is *N*-glycosylated. By contrast, when treated with endoglycosidase H (EndoH) that cleaves the β-1,4-bond between the two GlcNAc moieties of the N,N’ diacetylchitobiose core linked to the modified asparagine residue, a mobility shift was seen for the FLS2-YFP expressed in the pMAQ2 but not for the FLS2-YFP expressed in *cce2/cce3* background (Fig. 8c). Hence, the resistance to EndoH digestion shows that the *N*-glycan trees on FLS2-YFP occurring in the *cce2*/*cce3* background have a different structure from that in the parental pMAQ2 background.

To exclude misinterpretation due to overexpression used for most of the above assays, we used anti-FLS2 to look at endogenous FLS2 levels in microsomal membrane protein preparations. While not as obvious as the YFP-tagged FLS2, a slight difference in mobility of the FLS2 band can be seen in the *cce2*/*cce3* background. Furthermore, FLS2 protein levels were slightly reduced in the *cce2/cce3* mutants (Fig. 8d). Longer exposure of the western blot revealed that substantial FLS2 is still expressed in the mutants, which would explain why these mutants have weakened response but are generally still responsive to flg22 (Fig. 2). Taken together, altered glycosylation led to a marginal reduction of FLS2 levels and presumably the attenuated responses to flg22. The same situation can be assumed for all the PRRs examined in this study, albeit it remains to be tested if altered glycosylation influences ligand binding in each individual case.

### MAMP-induced resistance is not affected in the *alg3* mutants

**Fig. 9:**
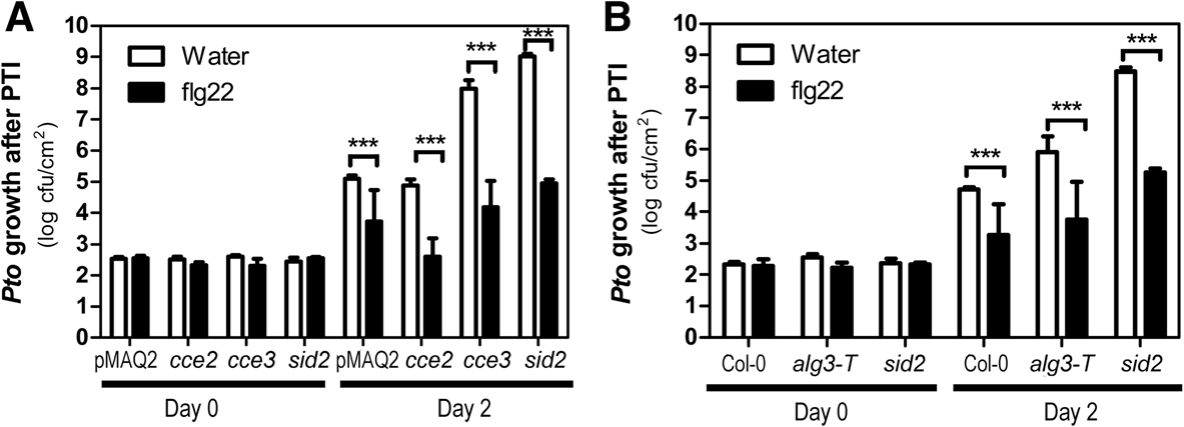
Pattern triggered immunity is not strongly affected in the *cce2*, *cce3* or *alg-T* mutants. **a**, **b** For the pattern-triggered immunity (PTI) assay, plants were infiltrated with 1 μM flg22 for 24 h before syringe-inoculation with *Pto* DC3000 (at 10^−5^ cfu/ml). Error bars depict standard errors (*n* = 6). Two-way ANOVA (with Bonferroni post-test) was used to evaluate statistical significance (*** = *p* < 0.001). The *sid2* mutant [47] was included as a hypersusceptible control but note that PTI appears to be intact in this mutant

Since altered glycosylation of PRRs is reflected by a reduced calcium response, we tested if MAMP-induced resistance to pathogens is affected. Flg22 pretreatment activates resistance to subsequent infection, leading to reduced growth of pathogens e.g. *Pseudomonas syringae* pv. *tomato* (*Pto*) DC3000 [46]. As seen in Fig. 9, this PTI response is maintained in the *cce2*, *cce3* and *alg3-T* mutants, as well as in the salicylic acid deficient *sid2* mutant [47], which was used as a reference for hypersusceptibility. Hence, in line with the calcium measurements showing reduced but still substantial responsiveness to MAMPs (Fig. 2), we may conclude that sufficient functional FLS2 (see Fig. 8d) is present to trigger the MAMP-induced resistance pathway. In addition to the induced resistance, note that the basal resistance levels between the three *alg3* mutants are different. While the *cce3* and *alg3-T* mutants (like *sid2*) supported more bacterial growth, the *cce2* plants are more resistant (see Additional file 1: Figure S3). These variations in susceptibility to *Pto* DC3000 may be due to additional mutations found in each mutant, especially for the EMS mutagenized *cce* mutants, and are unlikely to be linked to the *alg3* mutation. Taken together, while the basal resistance levels differ between the tested *alg3* alleles (due to still unknown reasons), the MAMP-induced resistance is mostly intact.

## Discussion

In this study, we show that *CCE2*/*CCE3* encodes ALG3, the α-1,3-mannosyltransferase that catalyzes the first mannosylation of Man_5_GlcNAc_2_-PP-Dol (M5^ER^) glycan precursors within the ER after the flipping of the glycan precursor from the cytosol to the ER luminal side [43–45]. In wild type plants, the resulting Man_6_GlcNAc_2_-PP-Dol is further mannosylated by two other ER-luminal mannosyltransferases, ALG9 and ALG12, to form the nine-mannose-containing Man_9_GlcNAc_2_-PP-Dol. The fully assembled glycan, Glc_3_Man_9_GlcNAc_2_, is formed subsequently through consecutive triglucosylation by ALG6, ALG8 and ALG10 glucosyltransferases, respectively (For a summary of the lipid-linked oligosaccharide biosynthesis pathway, see Additional file 1: Figure S1). With the aid of oligosaccharide transferase, the glycan is transferred to polypeptides for co- and post-translational *N*-glycosylation, which can affect folding and modulate the biological function of the proteins [48]. For instance, association with CNX or CRT lectins is important for the folding of nascent polypeptides and ER-quality control processes where ER-associated degradation (ERAD) pathway is activated if incomplete or misfolded glycoproteins are formed [49].

The *cce2* and *cce3* alleles lead to a premature stop (W139-) and an A63V exchange, respectively. While the shortened ALG3^W139-^ is non-functional, the ALG3^A63V^ variant can partially replace the missing function in *alg3* yeast but is apparently less efficient since it leads to the production of a mixture of fully assembled Glc_3_M_9_GlcNAc_2_ and M5^ER^ forms in the complemented yeast cells. Alternatively, since ALG3 is ER-localized in both yeast and Arabidopsis [43] where its action takes place, it is also possible that some of the ALG3^A63V^ may be partially mis-localized. Previous *N-*glycan profiling of total proteins from Arabidopsis *alg3* mutants showed rare *N-*glycan structures that are typically not detected; these include M3, M4^ER^, M5^ER^ and GlcM5^ER^ (see Additional file 1: Figure S1 for structures). There are also, overall, lower levels of complex-type *N-*glycan than in wild type Col-0 plants. Surprisingly, despite protein *N-*glycosylation differences compared with wild type, *alg3* showed no obvious phenotype under normal and high temperature or salt/osmotic stress conditions [44]. In an independent report where no high-mannose-type glycoproteins was detected in another *alg3* mutant (*alg3-2*, SALK_040296), no growth phenotype under normal growth conditions was also observed. However, the glycosylation abnormalities resulted in activation of marker genes diagnostic of an unfolded protein response. [43]. These results indicate that while AtALG3 is critical for mature *N-*glycosylation of proteins, it is not essential for cell viability and growth in Arabidopsis but may perhaps affect certain stress related processes. In accordance, our *cce2/cce3* mutants show normal growth (albeit the *cce2* is a little smaller in rosette size, not shown) but are compromised in the calcium response to multiple MAMPs/DAMPs. However, PTI resistance to subsequent bacterial infection is intact in the *cce2/cce3/alg3-T* mutants (Fig. 9). Alternatively, since some level of complex-type *N-*glycans is detectable in the *alg3* mutants, compensatory pathways to reduce impact of glycosylation defects probably exist in plants. One possibility is that after transport into the Golgi, the two α-1,2-mannose residues of M5^ER^ may be cleavable by α-1,2-mannosidase I; and the resultant M3 structure may be subsequently processed by *N-*acetylglucosaminyltransferase I (GnTI) and enable eventual maturation of plant-specific *N*-glycans in the Golgi apparatus [44]. Since GnTI has a 20-fold lower affinity for M3 compared to M5 [50], this reaction is feasible but perhaps less efficient. This “salvage” mechanism is indicative of the importance of proper *N-*glycosylation of proteins and hence viability of the organism.

In humans, N-glycosylation of the extracellular domain (ECD) of G protein-coupled receptors is necessary for functions ranging from agonist binding, folding, maturation, stability, to internalization [51]. Our work here showed that several PRRs are underglycosylated (Fig. 8) and contribute to attenuated MAMP/DAMP-induced calcium responses in the *cce2/cce3* (*alg3*) mutants (Fig. 2). In the human example mentioned above, the reduced localization of underglycosylated G-protein receptors to the cell surface may be, in part, compensated by the presence of receptor accessory proteins [51]. Whether members of the BAK1/SERK family that associate with FLS2 (or EFR or PEPR1) receptors may diminish the effects of underglycosylated plant PRRs remains to be tested. However, based on the failure to co-immunoprecipitate the ECDs of EFR and BAK1 after mutagenesis of a predicted *N*-glycosylation site (N590Q/S592T) in EFR, this does not seem to be the case – at least for EFR [52]. In another study where another conserved *N*-glycosylation site in the EFR ECD was mutated, this EFR^N143Q^ variant accumulated at reduced levels, lost the ability to bind elf18 ligand and the corresponding transgenic plants lacked elf18-elicited oxidative burst. This would explain why, despite EFR being correctly targeted to the plasma membrane, EFR function is impaired in *stt3a* mutants (encoding the STT3A subunit of the ER resident oligosaccharyltransferase, OST, complex) [31]. Glycosylation sites within the binding pocket may contribute to ligand binding in some cases (such as EFR) [31]. FLS2, on the other hand, tolerated mild underglycosylation occurring in *stt3a* mutants. These findings are corroborated in recent work using mutants of other OST subunits, *ost3* or *ost6*, where MAMP responses of EFR are more severely affected than the heavily glycosylated FLS2 [38]. Thus, distinct PRRs may be differentially affected by altered glycosylation. By contrast, we did not observe dramatic differences between the calcium responses to flg22 or elf18 in our *cce2/cce3* mutants, but this may be because ALG3 acts in an earlier step than the OST subunits of the *N*-glycosylation pathway (Additional file 1: Figure S1). Hence the difference may be PRRs modified with smaller glycan structures versus complete underglycosylation, respectively.

To the best of our knowledge, our work on ALG3 is the first report of such an early step of *N*-glycosylation on PRRs’ role in plant immunity signaling, while other studies are based on the later OST oligosaccharide transferase step. For plants, ALGs have, so far, mostly been implicated in growth and development. In EMS-mutagenesis screens for suppressor of the brassinosteroid *bri1-9* receptor dwarf mutant, *ASPARAGINE-LINKED GLYCOSYLATION-9* and −*12* (*ALG9/ALG12*) were isolated [53, 54]. These catalyze the two steps, after ALG3, of mannoysl-transfer to Man_6_GlcNAc_2_-PP-Dol in the ER (see Additional file 1: Figure S1). The *bri1-9* phenotype results from ERAD-mediated removal of the brassinosteroid receptors. Altered glycosylation caused by the *alg9* or *alg12* mutations affects ER quality control and ERAD of BRI1, thereby allowing – despite the *bri1-9* background – the accumulation of sufficient BRI1 receptors for brassinosteroid signaling. Surprisingly, although occurring in the same linear pathway, introducing the *alg3* mutation has the opposite effect (i.e. causing reduced accumulation) on BRI1-9 abundance [54]. Likewise, we see a marginal reduction of FLS2 levels in our *cce2/cce3* (*alg3*) mutants.

As discussed above, FLS2 may be more tolerant of alteration in glycosylation [38] and perhaps various PRRs are differentially affected. In our quantitative calcium measurements, the response to LPS seems proportionately more affected than the response to the other tested MAMPs/DAMPs in the *cce2*/*cce3* mutants (Fig. 2). The ECD of the putative LPS receptor, LORE [2], contains a B-type lectin-like S-domain that belongs to the S-locus domain initially described from gametophytic “Self-Incompatibility (SI)” locus receptors. Like the S-RNases associated with SI, which are known to be glycosylated within the putative recognition sites, it is likely that the LORE ECD may also be glycosylated. Accordingly, we see an altered mobility of the LORE-GFP fusion protein in our *cce2*/*cce3* mutants (Fig. 8a). *N-*glycosylation ensures the proper and efficient subcellular trafficking of S-locus receptors to the plasma membrane [55]. However, LORE localization to the plasma membrane appears to be unaffected in the *cce2/cce3* mutants (albeit this is based on overexpression through the strong 35S promoter). Future studies may reveal if altered glycosylation of LORE may affect affinity for its ligand but unfortunately, LPS binding assay is still a challenging task currently.

Besides the host, *N*-glycosylation is also important for pathogens. Mutation of *ALG3* in the rice blast fungus *Magnaporthe oryzae* causes underglycosylation and reduces the stability and chitin binding activity of the secreted SLP1 effector protein. SLP1’s inhibitory effect on chitin recognition by the rice chitin elicitor binding protein, CEBiP, is attenuated, resulting in a stronger immune response induction than that caused by wild type fungus. Consequently, the *M. oryzae alg3* mutant is less virulent [56]. We show here that ALG3 is important for proper PRR glycosylation and compromises plant immunity-related signaling such as calcium fluxes. Interestingly, *N*-glycosylation directly affects the surface expression and function of low-voltage-activated T-type calcium channels and control neuron excitability during glucose stimulation [57]. Since most membrane proteins will have to pass through the secretory pathway to target the plasma membrane, it is tempting to speculate that the still elusive plant calcium channel(s) responsive to MAMPs/DAMPs might perhaps also be glycosylated and affected by the *alg3* mutation. Finally, it should be mentioned that in animals/humans, *N*-glycosylation defects are often associated with diseases and especially congenital disorder [58]. Hence, *N*-glycosylation is highly conserved in eukaryotes. The lack of dramatic phenotypes for plant glycosylation mutants may be due to compensatory pathways to repair any deleterious effects of this important co- and post-translational protein modification pathway.

## Conclusions

The calcium signaling mutants, *cce2/cce3* are affected in *ALG3* (*Asparagine-linked glycosylation 3*), encoding the α-1,3-mannosyltransferase responsible for the first step of core oligosaccharide glycan assembly in the E.R. Defective glycosylation of exported proteins (e.g. MAMP receptors) in *alg3* mutants compromises immunity responses to MAMPs in plants. On the pathogen side, it has also been reported to affect virulence of fungal pathogens.

## Methods

### Plant material and growth

The *alg3-T* (SALK_064006, insertion in exon 6) and *alg3-T2* (SALK_046061, insertion in exon 3) lines have been described previously [44]. For calcium and MAPK assays, seeds were surface-sterilized, stratified at 4 °C for >2 d and grown in liquid MS under long day conditions (16 h light, 8 h dark cycles) as described [39]. For adult plants, seedlings were transferred to soil and grown in climate chambers under short day conditions (8 h light, 16 h dark cycles) at 22 °C.

### Mapping and identification of the mutation

The *cce2* mutant was crossed to the Arabidopsis accession Landsberg *erecta* (L*er*-0) and mapping performed as described [24]. Genomic DNA was prepared from young flower buds and sent to GATC Biotech (Constance, Germany) for Illumina whole genome sequencing.

Putative SNPs in *cce2* were validated by amplifying the corresponding genomic fragments from *cce2*, *cce*3 and pMAQ2 plants with the primers listed in Additional file 3: Table S2. The amplified fragments were sent for Sanger sequencing to GATC Biotech. Sequence alignments were performed with BioEdit (Tom Hall, Ibis Biosciences, Carlsbad, California, USA).

### Yeast complementation assays

The following yeast strains were used in this study: YG170 (*MATα ade2-101 ade3 ura3-52 his3 alg3-1 stt3-3*) and YG176 (*MATa ade2-101 ade3 ura3-52 his3Δ200 leu2 tyr1 stt3-3*) [59]. The *ALG3* ORF was amplified from cDNA prepared from pMAQ2, *cce2* or *cce3* plants (using primers listed in Additional file 3: Table S2) and cloned into *Bam*H I/*Xho* I-digested pYES2 vector (Invitrogen, Carlsbad, CA). All clones were verified by sequencing. The resulting pYES clones were introduced into YG170 strain by electroporation and complementation analysis carried out as described previously [44].

### Glycan analysis

Extraction of lipid-linked glycans from yeast, 2-pyridylamine labelling, size-fractionation HPLC and MS/MS analysis were performed as described previously [44].

### Protein extraction and western blotting

For protein extraction from mature plants or seedlings, frozen leaf discs or seedlings were homogenized in a Precellys 24 device (Bertin Technologies, Montigny-le-Bretonneux, France) in the presence of ~10–15 Zircosil® micro-milling beads (Ø1.2–1.7 mm) (Mühlmeier Mahltechnik, Bärnau, Germany) at 6500 rpm for 20 s. If necessary, the procedure was repeated until the tissues were thoroughly homogenized and to prevent premature thawing, samples were kept in liquid N_2_ between homogenization rounds. The transiently transformed protoplasts (in 1.5 ml tubes) were harvested by short pulse centrifugation in a Mikro120 microcentrifuge (Hettich, Tuttlingen, Germany), the incubation buffer was removed and the pellet was snap-frozen in liquid N_2_. Total protein was extracted from protoplasts or ground tissues by thawing the samples in 50 μL of extraction buffer (either Buffer A: 25 mM Tris–HCl pH 8.0, 150 mM NaCl, 1 % (v/v) 2-[2-[4-(2,4,4-trimethylpentan-2-yl)phenoxy]ethoxy]ethanol (Nonidet P-40), 1 % (v/v) Serva Protease Inhibitor mix P or Buffer B: 50 mM Tris pH 9.6, 100 mM NaCl, 20 mM DTT, 1 % (v/v) Serva protease inhibitor mix P, 0.1 % Triton X-100 and 0.1 % SDS) under constant vortexing for 15 min. Insoluble debris was pelleted by centrifugation at 20.000 x g for 10 min at 4 °C. Proteins were separated on 8 % SDS-PAGE gels and transferred onto PVDF membranes (Carl Roth, Karlsruhe, Germany). The blots were incubated with α-GFP (Life Technologies, Carlsbad, USA, 1:2000 dilution) in case of GFP- and YFP-tagged proteins or with α-FLS2 [60] for endogenous untagged FLS2 and the immune-decorated proteins detected with goat-anti-rabbit peroxidase-conjugated antibodies (Life Technologies, Carlsbad, USA) and enhanced chemiluminescence (ECL Prime, GE Healthcare, Freiburg, Germany).

### Deglycosylation assays

Total protein was extracted from protoplasts expressing FLS2-YFP as described above. Extracted proteins were boiled at 100 °C for 10 min in 1 x glycoprotein denaturing buffer (i.e. 0.5 % SDS, 40 mM DTT). Samples were allowed to cool to room temperature and 10 μL of each sample were either treated with 1 μL Peptide: N-glycosidase F in G7 reaction buffer (50 mM sodium phosphate pH 7.5, 1 % NP-40) or with 1 μL Endo H_f_ (Endoglycosidase H fused to maltose binding protein) in G5 reaction buffer (50 mM sodium citrate pH 5.5). Glycosidase treatment was performed for 1 h at 37 °C. All buffers and enzymes were obtained from New England Biolabs, Ipswich, Massachusetts, USA.

### Protoplast transfection and confocal microscopy

Protoplasts were isolated from 5-week-old plants from the pMAQ2/*cce1*/*cce2*/*cce3* genotype as described [61]. Protoplasts were transformed with pXCSG-FLS2-YFP, pXCSG-EFR-YFP [31], pAMPAT-PEPR-GFP, pUBC-CERK1-GFP (which was generated by LR-cloning recombination using a pDONR-CERK1 clone provided by Yusuke Saijo) or pGGInA-224C-LORE-eGFP (plasmid for 35S promoter-mediated expression of LORE-eGFP fusion; S.R., unpublished) [2]. After an overnight incubation to allow protein expression, microscopy of transformed protoplasts was performed with a LSM710 confocal laser-scanning microscope (Carl Zeiss, Jena, Germany). GFP signals were detected by excitation with a 488 nm argon laser and YFP molecules with a 514 nm argon laser. Images were acquired with the Zen 2012 software (Carl Zeiss) and processed with ImageJ software (National Institutes of Health, Bethesda, Maryland, USA) equipped with the Fiji plugin bundle [62] and the Biovoxxel toolbox (Jan Borcher/Biovoxxel; www.biovoxxel.de).

### Plant assays to assess responses to MAMPs (ROS, growth inhibition and pathoassays)

Detection of early MAMP-triggered responses such as MAPK activation and reactive oxygen species (ROS) accumulation and growth inhibition assays were performed as previously described [27].

### Availability of data

The datasets supporting the conclusions of this article are available in the LabArchives repository (https://mynotebook.labarchives.com/share/JLee%2520IPB%2520lab/MzUuMXwxNDcxNTUvMjcvVHJlZU5vZGUvMjY2OTg4MzE2OHw4OS4x) or DOI: 10.6070/H4RX9932. This includes (1) the numerical datasets used to plot Figs. 1, 2, 3, 4, 5, 6, 7, 8 and 9 and (2) the SNP/Indel lists generated from the Illumina Whole Genome Re-sequencing Data.

## Additional files

Additional file 1: Figure S1.
*N*-linked glycosylation model in yeast (A) and the *N*-linked glycan structures mentioned in the text (B). (A) Model is redrawn after Aebi [35] and Kajiura et al. [44]; and the *Saccharomyces cerevisiae* names of the enzymes or subunits are used. (B) The glycan structures mentioned in the text are schematically drawn using the same codes depicted in (A) above, which are based on the recommended Consortium for Functional Glycomics glycan structure nomenclature (http://www.functionalglycomics.org/static/consortium/Nomenclature.shtml). **Figure S2**: MAMP-induced seedling arrest assay. Representative photos showing root lengths of Arabidopsis seedlings grown on agar plates with or without flg22 (1 μM) for 14 days. **Figure S3**: Resistance to *Pseudomonas syringae* pv. *tomato* DC3000 (*Pto* DC3000) in the *cce2*, *cce3* or *alg3-T* mutants. A representative experiment (of three independently performed experiments) showing quantification of *Pto* DC3000 colony forming units (cfu) in leaf tissues after spray inoculation. Different alphabets indicate the distinct statistical groupings according to one-way ANOVA with Bonferroni’s multiple comparison test (*p* < 0.05). The hypersusceptible *sid2* mutant [47] was used as a control. (PDF 416 kb) Additional file 2: Table S1. List of single nucleotide polymorphisms (SNPs) found in the *cce2* mutant after Illumina-based genome resequencing. (XLSX 163 kb) Additional file 3: Table S2. Primers used in this work. (PDF 94 kb)

## Abbreviations

ACA8: arabidopsis-autoinhibited Ca^2+^-ATPase, isoform 8
ACT1: actin 1
ALG: asparagine-linked glycosylation
ALG3: asparagine-linked glycosylation 3
BAK1: BRI1-associated receptor kinase 1
BIR1: BAK1-interacting receptor-like kinase 1
BRI1: brassinosteroid insensitive 1
BSK1: brassinosteroid receptor-signaling kinase1
*cce*: *changed calcium elevation*
CERK1: chitin elicitor receptor kinase 1
CNGC2: cyclic nucleotide gated channel 2
CNX: calnexin
CPK5: calcium-dependent protein kinase 5
CRT: calreticulin
CYC1TT: cytochrome c terminator
DAMP: damage-associated molecular pattern
Dol-PP: dolichol-pyrophosphate
DPM1: dolichol phosphate mannose synthase 1
ECD: extracellular domain
ECL: enhanced chemiluminescence
EF1-α: elongation factor 1-α
EFR: EF-TU receptor
EF-Tu: elongation factor thermo unstable
EGF: epidermal growth factor
elf18: 18-residue peptide from EF-TU
EMS: ethane methylsulfonate
Endo H: endoglycosidase H
ERAD: ER-associated degradation
flg22: 22-residue peptide from flagellin
FLS2: flagellin sensitive 2
G.U.: glucose oligomer unit
GAL1pro: galactose-inducible yeast promotor
GFP: green fluorescent protein
Glc: glucose
GlcM5^ER^: unusual glycan structure, monoglucosylated M5^ER^
GlcN: glucosamine
GlcNAc: N-Acetylglucosamine
GnTI: N-acetylglucosaminyltransferase
HexNAc: hexose-acetylgucosamine structure
LIK1: LysM RLK1-interacting kinase 1
LPS: lipopolysaccharide
LRR: leucine-rich repeat
LYK5: LysM-containing receptor-like kinase 5
LysM: lysin motif
M3: glycan synthesis intermediate with 3 mannose residues, product of ALG2
M4^ER^: glycan synthesis intermediate with 4 mannose residues, product of ALG11
M5: trimmed glycan tree with 5 mannose residues, reaction catalyzed in Golgi apparatus
M5^ER^: glycan synthesis intermediate with 5 mannose residues, product of ALG11
MAMP: microbe-associated molecular pattern
Man: mannose
MAPK/MPK: mitogen activated protein kinase
MS: murashige and skoog
OST: oligosaccharyltransferase
PA: 2-pyridylamine
PAMP: pathogen-associated molecular pattern
PBL: avrPphB sensitive 1-like
PEP: *Arabidopsis thaliana* peptide
PEPR1: PEP1 receptor 1
PNGaseF: peptide-*N*-glycosidase F
PRR: pattern recognition receptor
*Ps* H4: *Pseudomonas aeruginosa* strain H4
PTI: pattern-triggered immunity
*Pto*: *Pseudomonas syringae* pathovar *tomato*
pv: pathovar
RBOHD: respiratory burst oxidase homolog D
RLCK: receptor-like cytoplasmic kinase
RLK: receptor-like kinase
RLP: receptor-like protein
RLU: relative light unit
ROS: reactive oxygen species
SEC59: secretory 59
SERK: somatic embryogenesis receptor kinase
SI: self incompatibility
*sid2*: *salicylic acid induction deficient 2*
SLP1: secreted lysM protein1
SNP: single nucleotide polymorphism
SOBIR1: suppressor of BIR1
S-RNAse: S-locus encoded ribonuclease
STT3A: staurosporin and temperature sensitive 3-like A
YFP: yellow fluorescent protein
α-FLS2: anti-FLS2
α-GFP: anti-GFP
