# Supplementary material for: Altered glycosylation of exported proteins, including surface immune receptors, compromises calcium and downstream signaling responses to microbe-associated molecular patterns in Arabidopsis thaliana
Source: BMC Plant Biol. 2016 Jan 28;16:31. doi: 10.1186/s12870-016-0718-3 (PMC4730752; doi:10.1186/s12870-016-0718-3)
Supplement: Additional file 1: Figure S1. — N-linked glycosylation model in yeast (A) and the N-linked glycan structures mentioned in the text (B). (A) Model is redrawn after Aebi [35] and Kajiura et al. [44]; and the Saccharomyces cerevisiae names of the enzymes or subunits are used. (B) The glycan structures mentioned in the text are schematically drawn using the same codes depicted in (A) above, which are based on the recommended Consortium for Functional Glycomics glycan structure nomenclature (http://www.functionalglycomics.org/static/consortium/Nomenclature.shtml). Figure S2: MAMP-induced seedling arrest assay. Representative photos showing root lengths of Arabidopsis seedlings grown on agar plates with or without flg22 (1 μM) for 14 days. Figure S3: Resistance to Pseudomonas syringae pv. tomato DC3000 (Pto DC3000) in the cce2, cce3 or alg3-T mutants. A representative experiment (of three independently performed experiments) showing quantification of Pto DC3000 colony forming units (cfu) in leaf tissues after spray inoculation. Different alphabets indicate the distinct statistical groupings according to one-way ANOVA with Bonferroni’s multiple comparison test (p < 0.05). The hypersusceptible sid2 mutant [47] was used as a control. (PDF 416 kb) [file 12870_2016_718_MOESM1_ESM.pdf]

Trempel *et al*. Additional File 1

Figure S1: *N*-linked glycosylation model in yeast and the *N*-linked glycan structures mentioned in the text.

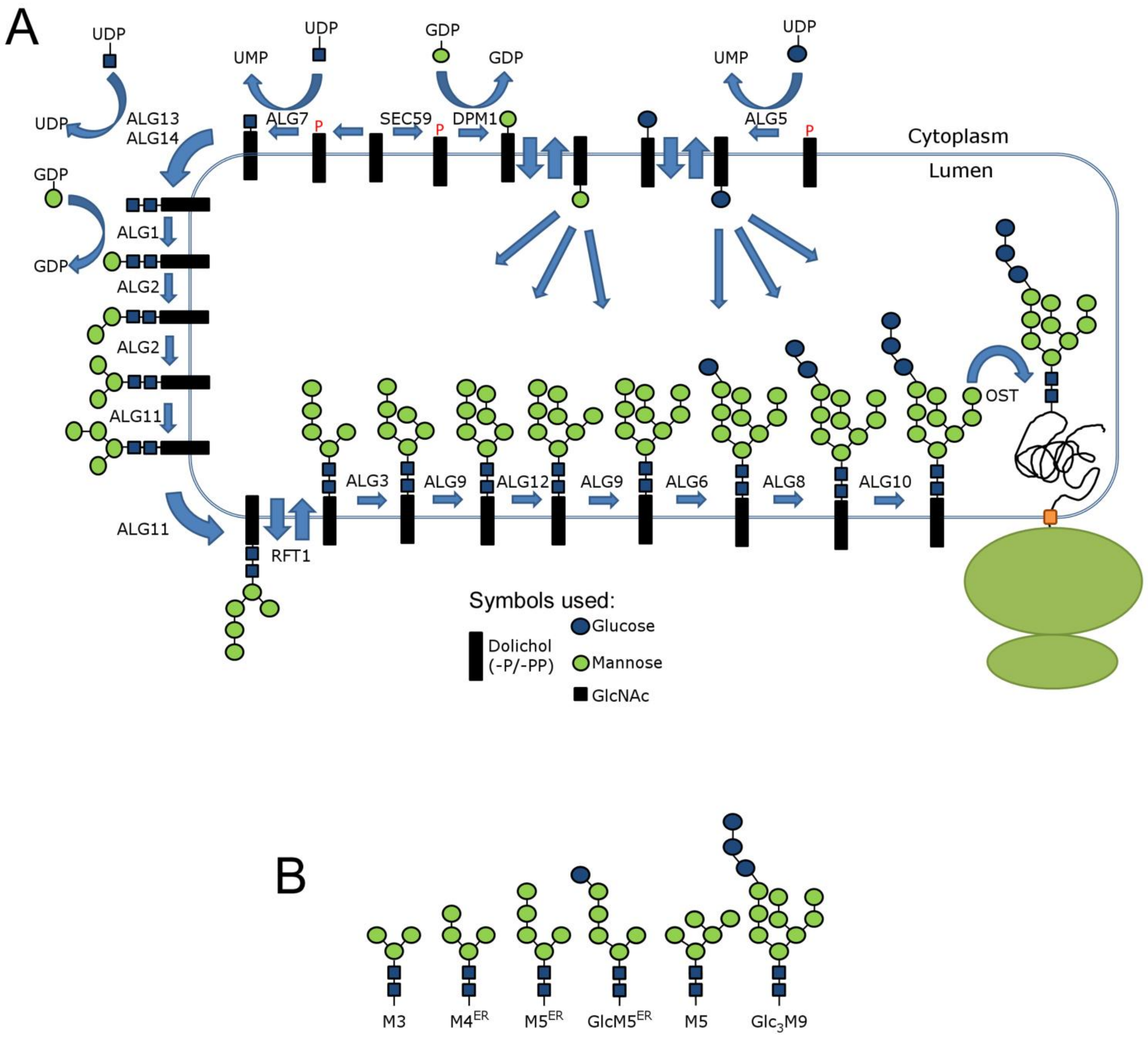

(A) The core oligosaccharide is preassembled on a dolichol anchor. Synthesis starts at the cytoplasmic side of the ER membrane, where glycosyltranferases (yeast loci *ALG 1,2,5,7,11,13,14*) involved in these first steps of synthesis have access to their soluble nucleotide sugar substrates. They assemble a structure composed of two *N*-acetylglucosamine and five mannose residues $M5^{ER}$. By the action of a flippase, the glycan is transferred to the luminal side of the ER membrane, where synthesis of the core oligosaccharide continues. Glycosyltransferases of the ER lumen are membrane spanning and use lipid-bound saccharides as their substrates (*ALG 3, 9, 12, 6, 8 and 10*). The assembled core oligosaccharide Glc_3M9, consisting of two *N*-acetylglucosamine, nine mannose and three glucose residues, is then transferred co- or posttranslationally to selected asparagine residues of a polypeptide by the Oligosaccharyltransferase complex (OST). Model is redrawn after Aebi, 2013 and Kajiura *et al.*, 2010; and the *Saccharomyces cerevisiae* names of the enzymes or subunits are used.
(B) The glycan structures mentioned in the text are schematically drawn using the same codes depicted in (A) above, which are based on the recommended Consortium for Functional Glycomics glycan structure nomenclature (http://www.functionalglycomics.org/static/consortium/Nomenclature.shtml).

Suppl. Fig. S2: MAMP-induced seedling arrest assay.

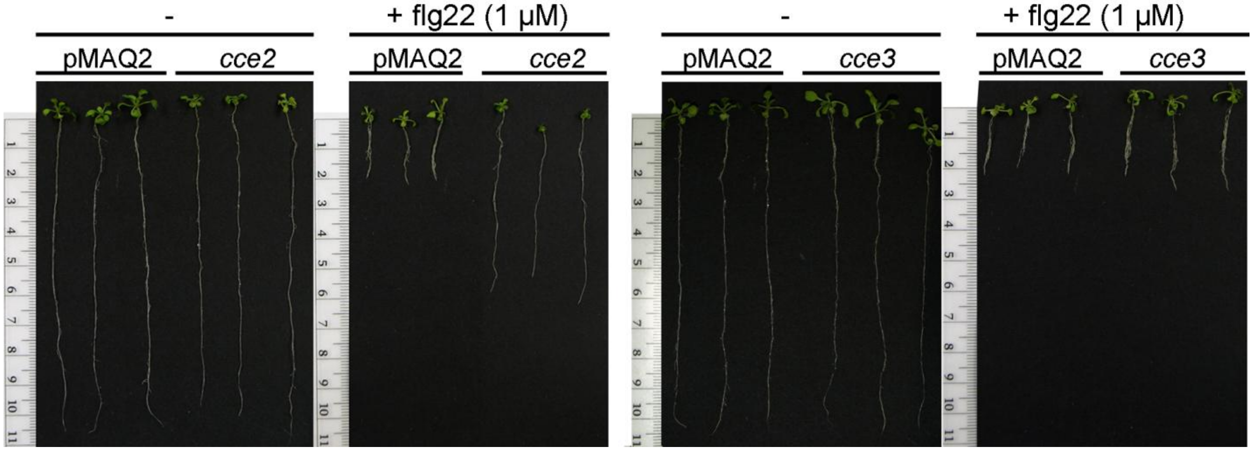

Representative photos showing root lengths of Arabidopsis seedlings grown on agar plates with or without flg22 (1 µM) for 14 days.

Suppl. Fig. S3: Resistance to *Pseudomonas syringae* pv. *tomato* DC3000 (*Pto* DC3000) in the *cce2, cce3* or *alg3-T* mutants.

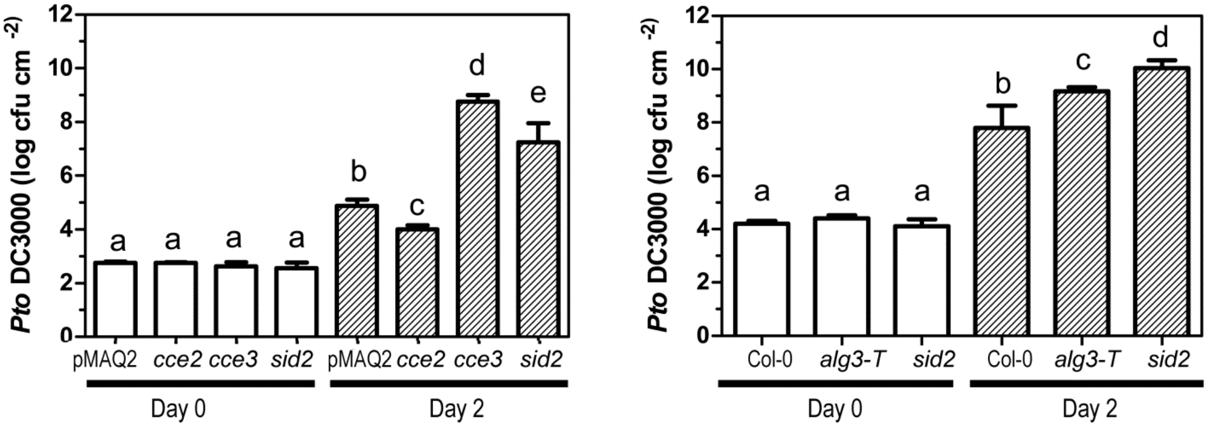

A representative experiment (of three independently performed experiments) showing quantification of *Pto* DC3000 colony forming units (cfu) in leaf tissues after spray inoculation. Different alphabets indicate the distinct statistical groupings according to one-way ANOVA with Bonferroni's multiple comparison test ($p < 0.05$). The hypersusceptible *sid2* mutant [47] was used as a control.
