# Supplementary material for: Altered glycosylation of exported proteins, including surface immune receptors, compromises calcium and downstream signaling responses to microbe-associated molecular patterns in Arabidopsis thaliana
Source: BMC Plant Biol. 2016 Jan 28;16:31. doi: 10.1186/s12870-016-0718-3 (PMC4730752; doi:10.1186/s12870-016-0718-3)
Supplement: Additional file 3: Table S2. — Primers used in this work. (PDF 94 kb) [file 12870_2016_718_MOESM3_ESM.pdf]

Table S2: Primers used in this work

Gene/AGI code	Primer	Sequence (5'→ 3')	used for	sqPCR cycle #
At2G46060	AT2G46060-1-F	CAAGTTGAAAGTGAGGTTCAGA	PCR	
At2G46060	AT2G46060-1-R	TTGTGGGCAGCAAATCTACA	PCR	
At2G46060	SAT2G46060-1-1	AAGGGAGAGAATATTTACCCATCA	sequencing	
At2G46060	SAT2G46060-1-2	TGATGCAGCTCTCAACTACCC	sequencing	
At2G46060	AT2G46060-2-F	AAGTTGCTGACGATGGGTTC	PCR	
At2G46060	AT2G46060-2-R	CGAAAATTCTCTCGAGTGATGA	PCR	
At2G46060	SAT2G46060-2-1	CTCGAGCCTGAAAGTGAAGC	sequencing	
At2G46060	SAT2G46060-2-2	ACCATTTTCCCCTGAGTCCT	sequencing	
At2G46060	AT2G46060-3-F	AATCGGCGTAAGTCAGGAAA	PCR	
At2G46060	AT2G46060-3-R	TCACAGCTTTATTGGCCTTG	PCR	
At2G46060	SAT2G46060-3-1	CCTGTCGAGCATGTTCAATG	sequencing	
At2G46060	AT2G46060-4-1-F	CTTGCGCTGATTCTTTTCCT	PCR	
At2G46060	AT2G46060-4-1-R	TCAAAAGCATATCGGCATTG	PCR	
At2G46060	SAT2G46060-4-F	CGAAAGCTGGTCGATGGTAT	sequencing	
At2G46060	SAT2G46060-4-R	ATGAGCTGCCTTAACCCAAA	sequencing	
At2G47760	AT2G47760-1-F	GGACGATTGCGATGCTTATT	PCR	
At2G47760	AT2G47760-1-R	TCACTCCCATCATAAACAGACAA	PCR	
At2G47760	SAT2G47760-1	CAAGAAGCCCAATAGGCTCA	sequencing	
At2G47760	AT2G47760-2-F	TTGCAGATTCTTTTTGGTGTTC	PCR	
At2G47760	AT2G47760-2-R	TTTAGATTTGGCGGGAGAGA	PCR	
At2G47760	SAT2G47760-2	GATCATCTACGTGAAGACTGATGTG	sequencing	
At2G47760	AT2G47760-3-F	GGGACTGCCGTTTCTGATAA	PCR	
At2G47760	AT2G47760-3-R	AATTGCCGCCAAATTACAAA	PCR	
At2G47760	SAT2G47760-3	GCCTTTGATCTTGGACGTGT	sequencing	
At2G47760	ALG3-GTWY-R2	TTATGCTTTTTTGTGTATTTGGGATTTAGGGTGTT	cloning	
At2G47760	ALG3-GTWY-F_a	CACCATGGCGGGCGCCTCATC	cloning	
At2G47760	alg3-4 F	ATGCAATCCTAGTCGCACTTATTA	verification of crossings with T-DNA	

			lines	
At2G47760	alg3-5 R	GATTGACAATGTAGAGAACACCAAAAA	verification of crossings with T-DNA lines	
At2G47760	alg3-6 F	TCTCTTTAATGATTGTTTTGCCAT	verification of crossings with T-DNA lines	
At2G47760	alg3-7 R	ATCAAAGGCGTTTGCTATGTATGAAA	verification of crossings with T-DNA lines	
-	LBd1	CCACGTTCTTTAATAGTGGACT	verification of crossings with T-DNA lines	
At2G47760	ALG3 semiQ F	TGCTGATGCAATCCTAGTCG	sqRT-PCR	30
At2G47760	ALG semiQ R	TAGTGCAGAGAGCGAGCAAA	sqRT-PCR	30
At1G07940	EF1A-s	TCACATCAACATTGTGGTCATTGGC	sqRT-PCR	25
At1G07940	EF1A-as	TTGATCTGGTCAAGAGCCTCAAG	sqRT-PCR	25
At2G47760	AtALG3-Fw	ATTGGATCCATGGCGGGCGCCTCATCACCG	RT-PCR, *Bam*HI site for cloning into pYES2	30
At2G47760	AtALG3-Re	ATTCTCGAGTTATGCTTTTTTGTGTATTTG	RT-PCR, *Xho*I site for cloning into pYES2	30
S. cerevisiae ACT1	ACT1-Fw	AGAGTTGCCCCAGAAGAACA	RT-PCR	30
*S. cerevisia*e ACT1	ACT1-Re	GGCTTGGATGGAAACGTAGA	RT-PCR	30
